# Preschoolers’ Sensitivity to Negative and Positive Emotional Facial Expressions: An ERP Study

**DOI:** 10.3389/fpsyg.2022.828066

**Published:** 2022-05-31

**Authors:** Sandra Naumann, Mareike Bayer, Isabel Dziobek

**Affiliations:** ^1^Berlin School of Mind and Brain, Humboldt-Universität zu Berlin, Berlin, Germany; ^2^Department of Psychology, Institute of Life Sciences, Humboldt-Universität zu Berlin, Berlin, Germany

**Keywords:** ERP, facial expressions, emotion processing, socio-emotional abilities, preschool age (3-5 years), developing brain

## Abstract

The study examined processing differences for facial expressions (happy, angry, or neutral) and their repetition with early (P1, N170) and late (P3) event-related potentials (ERPs) in young children (*N* = 33). EEG was recorded while children observed sequentially presented pairs of facial expressions, which were either the same (repeated trials) or differed in their emotion (novel trials). We also correlated ERP amplitude differences with parental and child measures of socio-emotional competence (emotion recognition, empathy). P1 amplitudes were increased for angry and happy as compared to neutral expressions. We also detected larger P3 amplitudes for angry expressions as compared to happy or neutral expressions. Repetition effects were evident at early and late processing stages marked by reduced P1 amplitudes for repeated vs. novel happy expressions, but enhanced P3 amplitudes for repeated vs. novel facial expressions. N170 amplitudes were neither modulated by facial expressions nor their repetition. None of the repetition effects were associated with measures of socio-emotional competence. Taken together, negative facial expressions led to increased neural activations in early and later processing stages, indicative of enhanced saliency to potential threating stimuli in young children. Processing of repeated facial expression seem to be differential for early and late neural stages: Reduced activation was detected at early neural processing stages particularly for happy faces, indicative of effective processing for an emotion, which is most familiar within this age range. Contrary to our hypothesis, enhanced activity for repeated vs. novel expression independent of a particular emotion were detected at later processing stages, which may be linked to the creation of new memory traces. Early and late repetition effects are discussed in light of developmental and perceptual differences as well as task-specific load.

## Introduction

During preschool age (3 to 5 years), children are increasingly exposed to opportunities for social learning, which is crucial for their socio-emotional development (Denham, 2006). One key facet of emotional competence constitutes the ability to recognize different facial expressions, which is particularly important for communicating effectively with others (Denham, 2018). Emotion recognition from facial expressions follows different developmental trajectories (Gao and Maurer, 2010). Whereas young children seem to detect happy expressions with almost adult-like precision, they are less accurate for negative emotions, such as anger or fear (Durand et al., 2007; Gao and Maurer, 2010). The relationship of quantitative and qualitative disparities in the development of emotion processing abilities during childhood remains a controversial topic. Some studies state that emotion recognition accuracy increases with age due to the progressive refinement of emotion categories (Johnston et al., 2011). Another line of research assumes that there is only a quantitative emotion processing difference due to emerging general cognitive abilities (McKone et al., 2012). Thus, the representation of facial expression categories may be difficult to discern with behavioral measures alone. Therefore, brain correlates, such as event-related potentials (ERPs), are useful to examine how young children perceptually encode and represent facial expression categories on a neural level. The processing of facial expressions requires attentional resources which can be stimulus-driven (e.g., Yantis, 1993) or top-down modulated (e.g., Hopfinger et al., 2000). Differences in the allocation of attentional resources can be observed at different neurophysiological stages during the processing of facial expressions (for review see Schindler and Bublatzky, 2020).

Across development, ERPs have been shown to successfully map early and late facial expression processing differences. Whereas infants’ ERP waveforms seem to differ from adults’ ERP morphology (e.g., due to physiological differences of the head; Leppänen et al., 2007), preschool-aged children already show a range of ERP responses evident in the adult literature on facial expression processing (Dennis et al., 2009). Initial and automatic detection of facial features is associated with early, sensory ERP components, like the P1 and N170, peaking at 100 ms and 170 ms, respectively (Hinojosa et al., 2015; Ding et al., 2017). Since the low-level analysis of the face occurs at the P1 level, it has been shown to be influenced by a stimulus’ motivational value (Rossi et al., 2017), spatial attention (Eimer et al., 2002), and physical properties (Schindler et al., 2021). The N170 may be involved in the parallel and interactive processing of facial identity and expression (Hinojosa et al., 2015). In preschoolers, N170 and P1 ERP components were the most commonly reported neural responses to face and expressive face stimuli (Bhavnani et al., 2021). Subsequent in-depth face processing is associated with higher-order, later ERPs, such as the P3 component, typically observed after 300 ms (Luo et al., 2010) and emerge during both passive face viewing and explicit attention tasks (see for review Schindler and Bublatzky, 2020). Though findings are heterogeneous, the majority of studies using passive face viewing paradigms showed that, in comparison to neutral facial expressions, positive and negative facial expressions led to increases in amplitudes of early and late components in infancy (Xie et al., 2019), in preschool-aged (Vlamings et al., 2010; Curtis and Cicchetti, 2011) and school-aged children (Anokhin et al., 2010).

Another branch of research investigated the neural categorization of facial expressions, presenting the same expression several times. In adults, the repetition of an identical facial expression led to a reduction in ERP amplitudes (Campanella et al., 2002). The repeated observation of a facial expression may re-activate an existing memory trace (e.g., emotion category associated with this facial expression) and thus ease its neural processing (Mueller et al., 2020). As a shift from low to high face processing proficiency is observed from infancy to school age (Johnston et al., 2011; Watling and Damaskinou, 2018), young childhood seems to be a particular sensitive developmental period to establish refined facial expression categories. So far, however, facial expression categorization with repetition has not yet been investigated in young children. Studies investigating the repetition of facial identities reveal similar facilitating effects in infants, preschool-, and school-aged samples (Itier and Taylor, 2004; van Strien et al., 2011; Peykarjou et al., 2016; Lochy et al., 2020).

In order to examine neural differences in facial expression categorization in young children, we adapted an existing paradigm previously employed in infants to examine facial identity processing (Peykarjou et al., 2016). Within a delayed match-to-sample task, children saw two sequentially presented facial stimuli (hereafter: *Face 1* and *Face 2*) which were either identical or differed with regard to their facial expression (happy, angry, or neutral). Subsequently, children had to indicate whether Face 1 and Face 2 displayed the same or a different emotion. As compared to previous paradigms which integrated a repetition condition (e.g., He and Johnson, 2018), the experimental design included longer than usual stimulus’ familiarization time to enable the creation of a reliable facial expression representation as well as shorter time between stimuli to reduce cognitive load (Peykarjou et al., 2016; Schweinberger and Neumann, 2016). Although most prominent results were reported with paradigms in which face stimuli were repeated in a highly frequent or long-lagged manner (Campanella et al., 2002; van Strien et al., 2011), we chose a paradigm that repeated stimuli immediately and only once, which has also been shown to elicit reduced activation (Peykarjou et al., 2016; Turano et al., 2017).

In similar facial expression matching tasks, children’s performance was better when facial expressions did not match, since non-matching expressions seem to be easier to detect than matching expressions (De Sonneville et al., 2002). Thus, we expected children to be faster and more accurate when Face 1 and Face 2 showed different facial expressions. Additionally, we expected the highest accuracy rates and fastest reaction times for pairings with happy expressions (De Sonneville et al., 2002). With regard to ERP responses, we predicted that amplitudes would be larger for emotional compared to neutral expressions. We expected happy expressions to elicit the largest amplitudes, followed by angry and neutral expressions (Curtis and Cicchetti, 2011; D'Hondt et al., 2017). Assuming that comprehensive facial expression representations are in place for young children, we expected an amplitude decrease in response to repeated facial expressions. Relative to angry or neutral expressions, we predicted that happy expressions would elicit the largest amplitude reduction because they are the most readily processed (Durand et al., 2007). In recent years, studies also reported links between early and late ERP responses to facial expressions and behavioral indexes of social–emotional processing (e.g., psychopathological traits; Kujawa et al., 2012; Hoyniak et al., 2019) and emotion regulation in preschool- and school-aged children (Dennis et al., 2009). Thus, we also associated parental and child measures of socio-emotional competence (emotion recognition and empathy) with ERP data to assess whether they were related to larger ERP sensitivity regarding repetition effects.

## Materials and Methods

### Participants

We estimated a sample size of 34 participants with G*Power (Faul et al., 2007), assuming a medium to large effect size of *f*^2^ = 0.25 for amplitude repetition effects (estimation based on experiment with similar paradigm of Peykarjou et al., 2016) and an attrition rate of 5% (similar to previous studies with young children: Gao and Maurer, 2010) for fixed effects in linear multiple regression to provide 80% power at a two-sided 5% α-level. The total sample consisted of 33 children aged 4 to 6 years. One participant was excluded for non-compliance during the EEG recording and one participant due to non-visibility of the ERP components (non-visibility due to below 10 trials per condition) leaving a final sample of 31 children (*M* = 5.14 years, SD = 0.67, 15 females). Participants were recruited from an existing university database. As compensation, families were paid € 16. All children showed normal intellectual functioning and receptive verbal ability as assessed by the Peabody Picture Vocabulary Test 4th Edition (PPVT-4; Dunn and Dunn, 2007) and Columbia Mental Maturity Scale (CMM; Eggert, 1972). We also screened for abnormalities in social ability with the Social Responsiveness Scale (SRS; Constantino and Gruber, 2005) and Social Communication Questionnaire (SCQ; Rutter et al., 2003). None of the children exceeded the cutoffs indicative of social impairments. Demographics included family income, caregiver occupation, and education, which were summarized to a socioeconomic score (SES; Winkler index; Winkler and Stolzenberg, 1998; range: 3–15, low SES = 3–6, medium SES = 7–10, high SES = 11–15). Families’ socioeconomic status ranged from middle to upper class. Screening and demographic information is described in Table 1.

**Table 1:** Participant demographics and characteristics.

Variables	Age (yrs)	SES	PPVT	CMM	STM	SRS	SCQ
Mean	5.14	11.77	58.46	60.13	44.68	35.03	4.26
SD	0.67	2.26	27.39	36.31	6.08	12.70	2.08

SES, Socioeconomic Status; PPVT, Peabody Picture Vocabulary Test; CMM, Columbia Mental Maturity Scale; STM, Short-term Memory; SRS, Social Responsiveness Scale; and SCQ, Social Communication Questionnaire.

The study protocol was reviewed and approved by the ethics committee of the Department of Psychology at Humboldt-Universität zu Berlin. The study was conducted in accordance with the Declaration of Helsinki. Informed consent for study participation was given by a parent prior to testing.

### Stimuli and Procedure

Stimuli consisted of happy, angry, and neutral facial expressions of 36 males and 36 females from standard face databases (Radboud Faces Database; Langner et al., 2010; Chicago Face Database; Ma et al., 2015). All face stimuli were grey-scaled and trimmed to the same oval shape to exclude hair and non-facial contours (height: 150 pixels, width: 110 pixels). Mean luminance levels were measured and adjusted for all stimuli. We calculated stimulus contrast values employing MATLAB R2016b (The MathWorks, Inc., Natick, MA, United States) toolboxes graycomatrix and graycoprops. We detected differences of contrast across emotion conditions [*F*(2,215) = 26.08, *p* < 0.001] with happy expressions having larger contrast values than angry (*p* < 0.001) or neutral expression (*p* < 0.001). Thus, to statistically control for low-level differences in stimulus contrast, we entered the individual stimulus contrast as additional covariate as well as stimulus as random intercept in all ERP analyses. Stimuli were presented on a grey background (RGB = 100, 100, 100) on a 15`` monitor (display resolution: 1024 × 767) that was positioned at a distance of approximately 70 cm from the participant (visual angle: 3.27°).

On the day of children’s testing, families were given a brief tour of the laboratory, received information about the testing, and were given the opportunity to ask questions. Thereafter, parents signed a consent form for their children’s participation in the study. During cap placement and recording, parents filled out questionnaires regarding their socioeconomic status and their children’s socio-emotional competences. Recording sessions took place in an electrically shielded and sound-attenuated booth. Children’s looking behavior was monitored using a video camera. One experimenter was seated next to the child during the testing to assist in directing its attention to the presentation screen.

**Figure 1:**
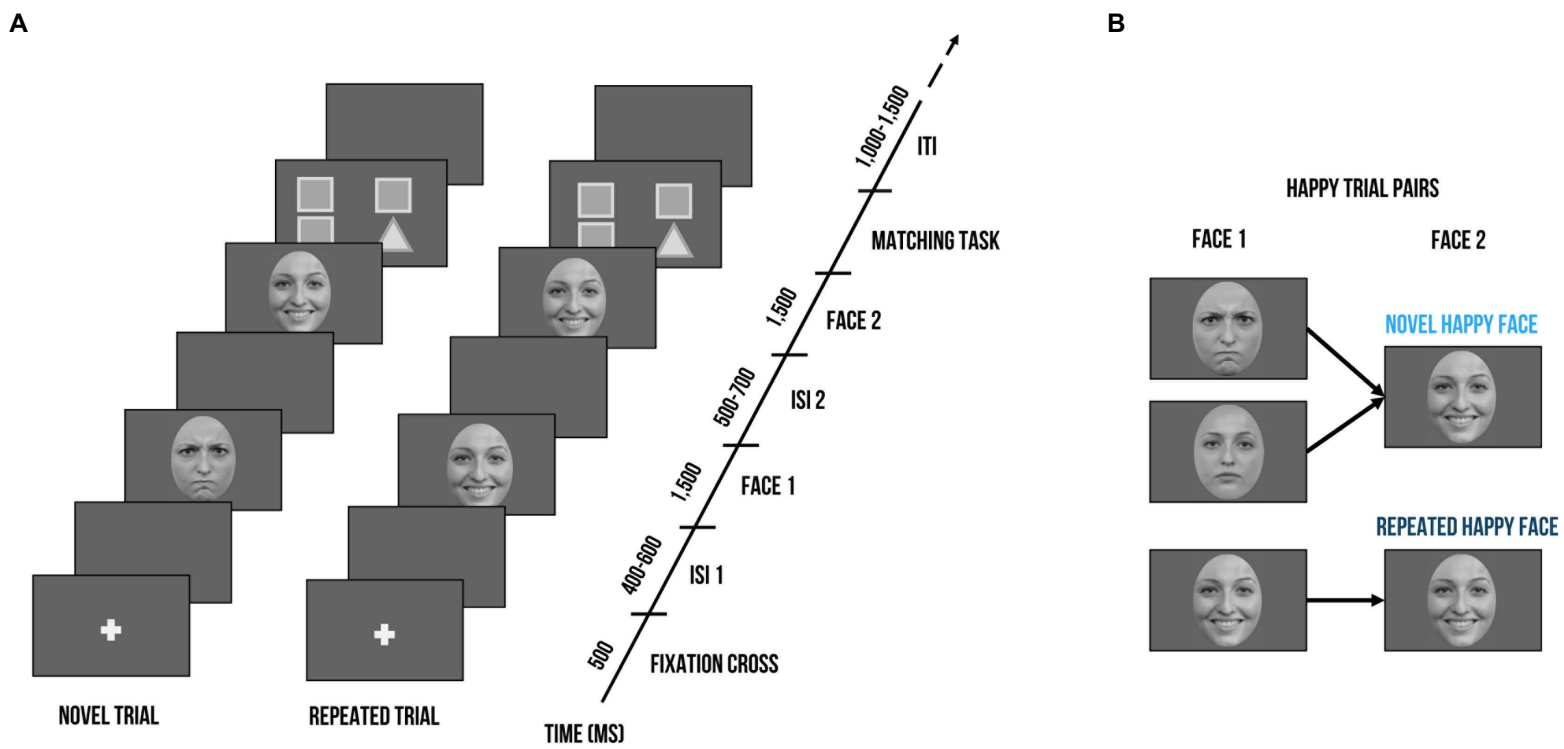
Visualization of the delayed match-to-sample task. **(A)** Exemplary trial sequence: Each trial included the presentation of a fixation cross (500 ms), followed by a blank screen (400–600 ms), Face 1 (1,500 ms), another blank screen (500–700 ms), and Face 2 (1,500 ms). Face 2 could either bear the same facial expression as Face 1 (*repeated trial*) or a different one (*novel trial*). After seeing Face 2, children had to indicate whether the facial expression of Face 1 and Face 2 were the same; triangles and squares shapes on screen served as reminders of the button order (e.g., two squares on the left side indicated that the left button needed to be pressed when the facial expression of Face 1 and Face 2 was repeated). **(B)** Exemplary trial pairs for happy expressions. *Novel* happy face trials started with a neutral or angry expressions at Face 1 and always contained a happy expression at Face 2. *Repeated* happy face trials consisted of the same happy facial expression at Face 1 and Face 2. Face stimuli are obtained from the Radboud Faces Database (Langner et al., 2010). The depicted individuals provided written informed consent for the publication of their identifiable images.

As shown in Figure 1, a trial consisted of two faces of the same identity (Face 1 and Face 2). Face 1 was either followed by a Face 2 with the same facial expression (repeated trial) or a different facial expression (novel trial). As an example, novel happy trials could either contain an angry or neutral expression as Face 1, but Face 2 was always a happy expression. In contrast, a repeated happy trial contained a happy expression at both Face 1 and Face 2. Each trial was set up as follows (Peykarjou et al., 2016): A fixation cross was presented for 500 ms, which was followed by a jittered inter-stimulus interval (ISI; 400–600 ms) and Face 1 (1,500 ms). After another jittered ISI (500–700 ms), Face 2 (1,500 ms) was presented. At the end of a trial, children had to indicate whether the facial expression of Face 1 and Face 2 matched. They had a small button box with one button in each hand. On screen, formation of triangles and squares served as reminders of the button order (e.g., two squares on the left side indicated that the button in the left hand needed to be pressed when the facial expression of Face 1 and Face 2 was repeated). The button order was counterbalanced. Between trials, there was a jittered inter-trial interval (ITI) of 1,000–1,500 ms. There were 3 blocks with 48 trials each, summing up to a total of 144 trials. Within blocks, no condition, gender, or valence was repeated more than three times successively. Face identity was never repeated within a block and maximally repeated twice throughout the whole paradigm. Face gender was equally distributed across blocks. For each valence (happy, angry, neutral), there were 16 trials per block, half of them repeated, which resulted in 24 repeated and 24 novel trials. Participants were instructed to keep their gaze at the center of the presentation screen. In order to guarantee that the children paid attention, an animal cartoon picture would appear randomly at the location of the face stimuli after some face trials. Participants were told that if they spotted all animals and pressed a button at their appearance, they would get to see all animals integrated into a nature scene after each block. A short practice session with 4 practice trials preceded the actual test session. During the EEG task, we also recorded reaction times and accuracy. The task was administered using Presentation^®^ software (Version 17.2, Neurobehavioral Systems, Inc., Berkeley, CA). After the recording session, children’s intellectual and verbal functioning as well as emotion recognition and empathic skills were assessed.

#### Emotion Recognition and Empathy Measures

We employed an emotion matching task (EMT; Watling and Damaskinou, 2018) using the facial stimuli of the delayed match-to-sample task. Children saw a pair of faces of the same identity but with a different facial expression (happy, angry, neutral). Both faces were presented at the same time. While faces were on screen, children heard a voice-over of an emotion word (happy, angry, or neutral). They had to indicate by button press which of the faces (left or right) matched the voice-over. Facial expressions were equally distributed as well as randomized and the button order was counterbalanced. The EMT served as a counterpart to the delayed match-to-sample task to examine whether children could correctly identify the different facial expressions and to provide an additional set of behavioral measures (reaction times and accuracy).

We assessed children’s emotion recognition and empathy skills with the *Inventory to survey of emotional competences for three- to six-year-olds* (EMK 3–6; Petermann and Gust, 2016). The EMK 3–6 shows good internal consistency (Cronbach’s *α* = 0.78–0.90) as well as validity (Gust et al., 2017). It includes a parental questionnaire and child assessments to evaluate socio-emotional competences. Children had to identify other children’s emotions on picture cards and explain why children might feel that particular way. As for the empathy task, they were asked to imagine themselves in emotionally charged situations through a doll’s perspective (e.g., the doll is afraid of dogs, what happens if the doll meets a dog?) and come up with coping strategies (e.g., to chase the dog away). Composite z-scores were calculated for parental ratings and child assessment.

#### Short-Term Memory Task

We included a short-term memory task to control for children’s general cognitive abilities, which required the child to recite numbers (Esser and Wyschkon, 2016). The examiner would name a sequence of numbers and ask the child to repeat them in the same order. The first sequence started with two numbers and progressed to up to nine numbers. The examiner stopped the task when the child was not able to recite the numbers after the examiner had repeated them twice. Points were administered for each round (2 points if child repeated numbers without help, 1 point if child needed to hear digits twice) and summed into one score. Sum scores where transformed into standardized scores by using children’s age.

### EEG Recording, Processing, and Analysis

EEG signals were collected with the QRefa Acquisition Software, Version 1.0 beta (MPI-CBS, Leipzig, Germany) from 46 Ag/AgCl electrodes attached to elastic caps (EasyCap GmbH, Germany) at standard positions and synchronized with the onset of stimulus presentation. Electrode impedances were kept below 10 kΩ. Digitalization of the EEG data was carried out continuously at a sampling rate of 500 Hz (anti-aliasing low-pass filter of 135 Hz). EEG recordings were referenced online to CZ with the ground electrode at FP1. Electro-oculograms were registered with electrodes at the outer canthi of both eyes and at the orbital ridge of the right eye.

Further offline pre-processing and analyses were carried out in MATLAB R2016b using EEGLAB (Delorme and Makeig, 2004) and the toolboxes SASICA (Chaumon et al., 2015) and ERPLAB (Lopez-Calderon and Luck, 2014). Data were high-pass filtered at 0.01 Hz and low-pass filtered at 30 Hz with an IIR Butterworth filter (2nd order) as well as a Parks-McClellan Notch filter at 50 Hz. EEG data were re-referenced to the average of all data channels (excluding eye channels) and segmented from 200 ms before stimulus onset to 1,500 ms post-stimulus onset. Baseline correction was based on the mean activity during the 200 ms prior stimulus onset. We manually removed non-systematic noise (e.g., pulling the cap) to improve the succeeding independent component analysis (ICA) for ocular artifact removal. SASICA was then used to mark and reject the detected ocular artifacts. Afterwards, segments that still contained artifacts were manually rejected on the base of a semi-automated artifact rejection with a voltage criterion (exceeding ± 200 μV) and visual inspection of each trial. After artifact rejection, the mean number of trials per condition was not significantly different for facial expressions [happy: *M* = 39.9, SD = 6.2, angry: *M* = 39.5, SD = 6.5, neutral: *M* = 39.4, SD = 5.9; *F*(2, 90) = 0.83, *p* = 0.44]. Trial numbers for repeated trials (*M* = 58.2, SD = 9.1) were significantly lower compared to novel trials [*M* = 60.6, SD = 9.0; *t*(30) = 5.0, *p* < 0.001]. Please note that further trials were removed within the statistical analysis due to reaction time exclusion criteria, for final trial numbers and statistics see below.

**Figure 2:**
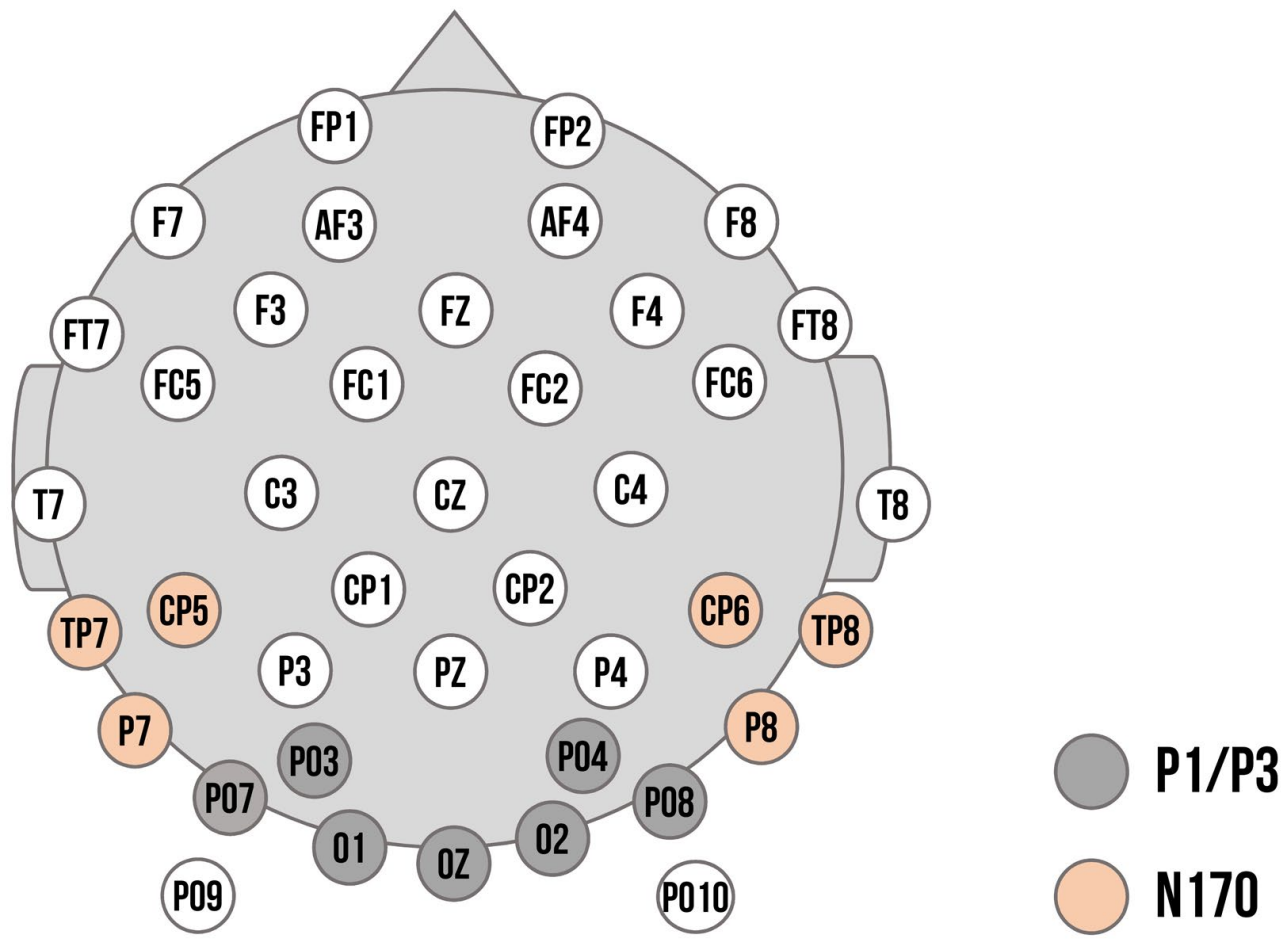
Electrode montage with channel locations used as regions of interest (ROIs). Dark grey: Channels used for the P1 and P3 component. Orange: Channels used for the left and right cluster for the N170 component.

Regions of interest for the ERP components and time windows were based on previous research (Batty and Taylor, 2006; Curtis and Cicchetti, 2011) as well as visual inspection of the ERP topographies averaged across all conditions and participants. Previously, P3 responses have been found to be maximal over parietal locations (e.g., Kujawa et al., 2012). Within our sample, however, visual inspection of topographies across all conditions showed maximal activation over more parieto-occipital sensors (previous studies with young children also chose sensors at more occipital or parietal-occipital sensors: e.g., Vlamings et al., 2010; Curtis and Cicchetti, 2011; MacNamara et al., 2016). Thus, ROIs for P1 and P3 were composed of the electrodes PO3, O1, PO7, Oz, O2, PO4, and PO8. In a parallel fashion, maximal N170 responses have been typically observed at parietal-occipital regions (e.g., Rossion and Jacques, 2008). Visual inspection showed slight diversion from the literature: we chose a left temporal–parietal cluster (P7, TP7, CP5) and a right temporal–parietal cluster (P8, TP8, CP6) to score the N170. The electrode layout is demonstrated in Figure 2. P1 and N170 peaks for each participant were identified using peak detection procedures and quantified as mean amplitude in a time window of 20 ms around the peak. P1 peaks were determined in the time window of 90 to 130 ms; N170 peaks in the time window of 180 to 220 ms. The P3 was quantified in the time window of 300 to 500 ms. Time windows are comparable to previous ERP studies examining preschool samples (e.g., Dawson et al., 2004; D'Hondt et al., 2017).

### Statistical Analyses

Statistical analyses were performed using R-Studio (R Core Team, 2020). For all analyses, we excluded trials with reaction times (RTs) lower than 250 ms (Johnston et al., 2011) and incorrect trials (Langeslag et al., 2020). Neither the final number of trials for each facial expression [happy: *M* = 33.5, SD = 6.7, angry: *M* = 32.7, SD = 7.3, neutral: *M* = 33.3, SD = 8.1; *F*(2, 87) = 0.1, *p* = 0.91] nor for repeated (*M* = 50.1, SD = 14.6) vs. novel trials (*M* = 49.4, SD = 13.7) differed significantly [*t*(29) = 0.2, *p* = 0.9].

All (general) linear mixed model analyses were conducted with the lme4 package (Bates et al., 2015). Assumptions for multiple regression were checked for all models (normality of the residuals, linearity, multicollinearity, homoscedasticity). Marginal and conditional $R^{2}$ were calculated as measures of goodness of fit for mixed models, in which marginal $R^{2}$ reflects variance explained by fixed factors, and conditional $R^{2}$ variance explained by the entire model. The *p*-values (uncorrected) were computed *via* Wald statistics approximation (treating *t* as Wald *z*); interaction effects were delineated with post-hoc tests (multcomp package; v1.4–16, Hothorn et al., 2008). As first fixed factor, we included a contrast for repetition (novel vs. repeated trials; contrast coding: [−0.5, 0.5]). Further, we defined two contrasts to compare effects between facial expressions: The first contrast disentangled the averaged effect of both emotional expressions compared to neutral expressions (emotional [average of happy/angry] vs. neutral expressions; contrast coding: [−0.25, −0.25, 0.5]), while the second contrast compared the effect of happy vs. angry expressions (contrast coding: [0.5, −0.5,0]). We also included the interaction between facial expressions and repetition contrasts as fixed factor. Short-term memory scores were entered as a scaled covariate in all (G) LMM analyses to control for cognitive task demands. As random intercepts, we included participant and facial stimulus.

For the delayed match-to-sample task, we calculated a general linear mixed model (GLMM) for accuracy rates and a linear mixed model (LMM) for RTs. Additionally, RTs were log-transformed (determined with the Box–Cox procedure; Box and Cox, 1964) to meet the assumption of normally distributed residuals. Regarding the ERP components, we focused on neural responses to Face 2 employing linear mixed models and including physical stimulus contrast as additional scaled covariate to control for low-level differences, as well as electrode as additional random intercept. As hemispheric differences were previously reported for the N170 component (Batty and Taylor, 2006), we also included hemisphere as fixed factor for the N170 analysis (left vs. right ROI). Results for Face 1 are reported in the supplement.

For the EMT, we only applied the facial expression contrast within a GLMM for accuracy rates and an LMM for RTs. We only report findings for short-term memory or stimulus contrast if found to be significant (see Supplementary Material for full model statistics). Lastly, we performed correlational analyses using Pearson’s correlations to associate brain and behavior variables. For significant ERP modulations by facial expression and repetition, we calculated amplitude difference scores between conditions and correlated them with the composite scores of empathy and emotion recognition. We used the false discovery rate (FDR) to correct for multiple comparisons in post-hoc tests and correlational analyses.

## Results

### Delayed Match-to-Sample Task Performance

**Figure 3:**
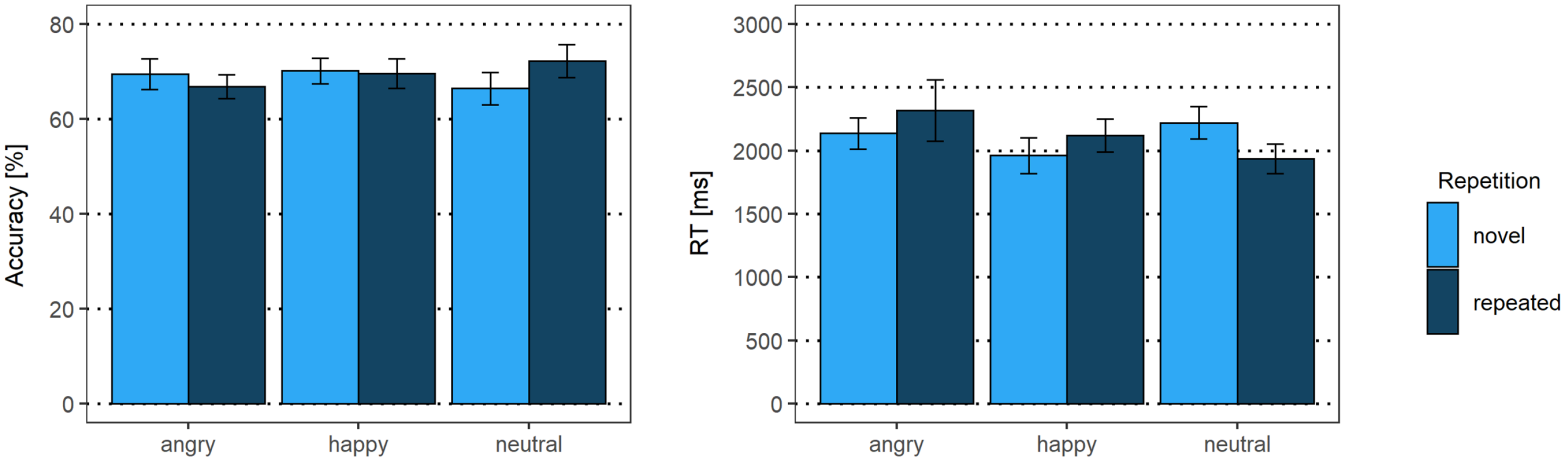
Accuracy rates and reaction times of the delayed match-to-sample task. Error bars indicate standard errors (SE).

As displayed in Figure 3, there were no accuracy differences for facial expression contrasts (emotional vs. neutral expressions: *β* < 0.01, *p* = 0.97, *OR* = 1.00 [95% CI: −0.65, 2.65]; happy vs. angry expressions: *β* = −0.17, *p* = 0.07, *OR* = 0.85 [95% CI: −0.55, 2.24]) or repetition (*β* = −0.01, *p* = 0.95, *OR* = 0.99 [95% CI: −0.64, 2.63]). Interactions between facial expression contrasts and repetition yielded no significant results (emotional vs. neutral expressions x repetition: *β* = 0.23, *p* = 0.29, *OR* = 1.25 [95% CI: −0.81, 3.32]; happy vs. angry expressions x repetition: *β* = 0.26, *p* = 0.17, *OR* = 1.30 [95% CI: −0.84, 3.43]).

For reaction times, the emotional vs. neutral facial expression contrast yielded no significant results (*β* = 0.05, *p* = 0.31). In line with our hypothesis, happy faces were detected faster than angry faces (*β* = 0.09, *p* = 0.03), indicating that the correct identification of a Face 2 with a happy expression as either repeated or novel was faster than for a Face 2 with an angry expression. The main effect for repetition was not significant (*β* = −0.02, *p* = 0.59). The interaction of emotional vs. neutral expressions with repetition, however, was significant (*β* = −0.20, *p* = 0.03). Novel happy faces were detected faster than novel neutral faces (*p* = 0.01), suggesting that, irrespective of what was presented as Face 1, when Face 2 showed a novel happy expression, it was faster identified correctly as novel compared to a novel neutral face. None of the other post-hoc tests were significant (all *p* > 0.75). Similarly, the interaction of happy vs. angry expressions with repetition was not significant (*β* = −0.11, *p* = 0.19; see Supplementary Tables S1, S2).

### ERP Responses

#### P1

**Figure 4:**
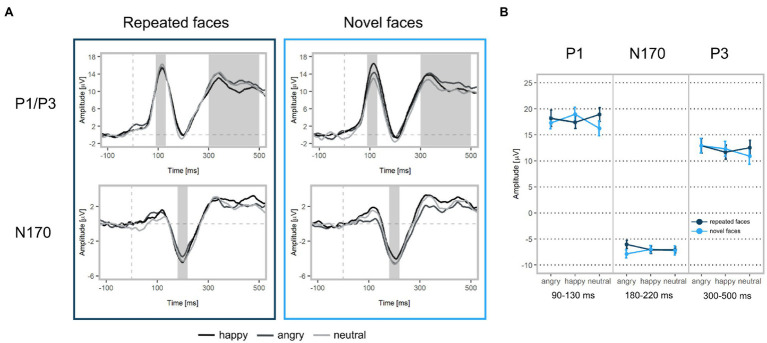
ERP waveforms and mean amplitudes at Face 2. **(A)** Upper left: Grand-averaged P1 and P3 waveforms for repeated happy (black), angry (dark grey), and neutral (light grey) facial expressions (ROI: PO3, PO4, PO7, PO8, O1, O2, Oz). Upper right: Grand-averaged P1 and P3 waveforms for novel facial expressions. Bottom left: Grand-averaged N170 waveforms for repeated happy, angry, and neutral facial expressions (ROI: P7, TP7, CP5, P8, TP8, CP6). Bottom right: Grand-averaged N170 waveforms for novel facial expressions. Shadowed areas indicate the time windows used to identify participants’ individual peaks and mean amplitudes. **(B)** Mean P1, N170, and P3 amplitudes and standard deviations separately for each condition.

**Figure 5:**
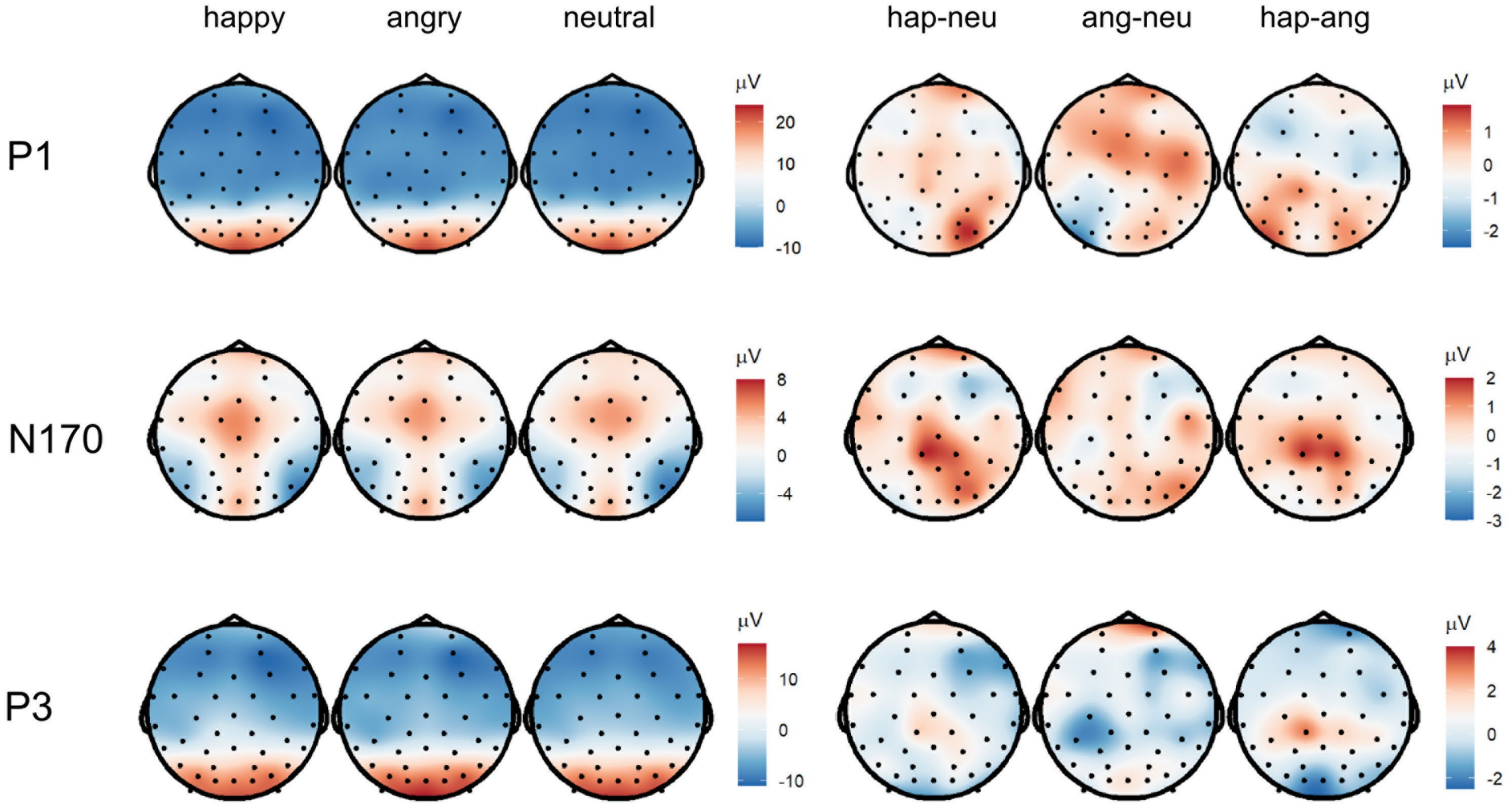
Topographies of the averaged P1 (90–130 ms), N170 (180–220 ms), and P3 (300–500 ms) activity displaying scalp topographies and difference topographies (in μV) for the emotion condition.

In line with our hypothesis, we found larger P1 amplitudes for emotional vs. neutral expressions (*β* = −1.29, *p* = 0.001; Figures 4, 5 as well as Supplementary Figure S1 of the Supplementary Material). Both happy (*p* = 0.003) and angry expressions (*p* = 0.03) elicited larger P1 amplitudes compared to neutral expressions. No amplitude differences were detected between happy and angry expressions (*β* = −0.27, *p* = 0.44). There was no main effect of repetition (*β* = −0.11, *p* = 0.73). Similarly, no interaction of emotional vs. neutral expressions with repetition was found (*β* = 1.20, *p* = 0.14). We did, however, detect a significant interaction of happy vs. angry expressions with repetition (*β* = 2.75, *p* < 0.001): Post-hoc tests indicated that, in line with our hypothesis, P1 amplitudes for repeated happy expressions were smaller than for novel happy expressions (*p* = 0.003). Novel happy expressions elicited larger P1 amplitudes than novel angry expressions (*p* = 0.005; all other *p* > 0.12; see Supplementary Tables S3–S5).

#### N170

We did not find significant main effects for facial expression contrasts (emotion vs. neutral expressions: *β* = −0.08, *p* = 0.83; happy vs. angry expressions: *β* = 0.11, *p* = 0.75) or repetition (*β* = 0.07, *p* = 0.80). None of the interactions of facial expressions with repetition were significant (emotion vs. neutral expressions: *β* = 0.87, *p* = 0.43; happy vs. angry expressions: *β* = 1.16, *p* = 0.08, see Supplementary Table S3).

#### P3

In line with our hypothesis, we detected differences for emotional vs. neutral facial expressions (*β* = −1.05, *p* = 0.02). Angry expressions elicited larger P3 amplitudes than neutral expressions (*p* = 0.001) and happy expressions (*β* = 1.10, *p* = 0.004). No significant difference was found for happy vs. neutral expressions (*p* = 0.77). In contrast with our hypothesis, we detected a significant main effect of repetition, indicating that repeated faces elicited larger P3 amplitudes than novel faces (*β* = 0.88, *p* = 0.014). Interactions of facial expression contrasts with repetition were not significant (emotional vs. neutral facial expressions x repetition: *β* = 0.73, *p* = 0.42; happy vs. angry facial expressions x repetition: *β* = −0.78, *p* = 0.32). Additionally, we detected that stimulus’ contrast was a significant covariate, with larger contrast values eliciting larger P3 amplitudes (*β* = −0.50, *p* = 0.03, see Supplementary Tables S3, S6). All ERP results for Face 1 (examining facial expression contrasts) are reported in the supplement (see Supplementary Tables S7–S9).

### Emotion Recognition and Empathy Measures

#### Emotion Matching Task Performance

There were no accuracy differences between emotional vs. neutral expressions (*β* = 0.10, *p* = 0.62, *OR* = 1.10 [95% CI: −0.71, 2.92]) or happy vs. angry expressions (*β* = 0.33, *p* = 0.05, *OR* = 1.39 [95% CI: −0.90, 3.69]). For reaction times, the emotional vs. neutral expression contrast was significant (*β* = 0.11, *p* = 0.01). Post-hoc tests indicated that happy expressions were detected faster than neutral expressions (*p* = 0.01; angry vs. neutral: *p* = 0.18). The happy vs. angry contrast yielded no significant results (*β* = 0.05, *p* = 0.19, see Supplementary Tables S10, S11).

#### ERP Associations With Socio-Emotional Competence Measures

We calculated difference scores of significant facial expression × repetition interactions (novel happy-repeated happy, novel happy-novel angry) for P1 amplitudes and a difference score for the P3 main effect of repetition (novel-repeated). Subsequently, we associated them with EMK 3–6 empathy and emotion recognition composite scores. None of the correlations of emotion recognition or empathy with P1 or P3 difference scores survived FDR correction (all *p* > 0.63, see Supplementary Tables S12, S13).

## Discussion

Our study sought to provide further evidence on young children’s neural representation of facial expressions. To this aim, we employed a delayed match-to-sample task in which two faces (Face 1, Face 2) of the same identity were presented in succession, either displaying the same (repeated trial) or a different (novel trial) facial expression. Subsequently, children were asked to indicate whether facial expressions of Face 1 and Face 2 matched. We assessed neural representations with ERPs of early (P1, N170) and late (P3) facial expression processing. Additionally, we examined reaction times and accuracy rates as well as associations with measures of socio-emotional competence (emotion recognition and empathy). In line with our hypothesis, we found that, independent of repetition, correct match/mismatch decisions were fastest when Face 2 was happy as compared to angry. For novel trials, correct match/mismatch decisions were also faster when happy vs. neutral expressions were presented as Face 2. Additionally, the EMT indicated that happy expressions were detected faster than neutral expressions. As hypothesized, modulations by expressions were found for early and late ERPs. However, no overall advantage of happy expression was apparent: P1 amplitudes were larger for angry and happy expressions as compared to neutral expressions. P3 amplitudes were larger for angry compared to happy and neutral expressions. Repetition effects were visible at early and late processing stages: We found reduced P1 amplitudes for repeated happy expressions as compared to novel happy expressions. Additionally, novel happy expressions elicited larger P1 amplitudes than novel angry expressions. In contrast to our expectations, we detected larger P3 amplitudes for repeated compared to novel facial expressions. None of the repetition effects were associated with measures of socio-emotional competence.

### Emotion Modulation in Early and Late ERPs

In line with our hypotheses, we detected larger amplitudes for emotional vs. neutral expressions in early and late ERP responses. Modulations by facial expressions, however, slightly diverted from what we had predicted. For early ERPs, we found larger P1 amplitudes for happy and angry as compared to neutral expressions, but no differences between happy and angry expressions (effect also detected at Face 1, see Supplementary Tables S7, S8). With reference to previous research, some studies showed P1 amplitude differences between happy and angry expressions (D'Hondt et al., 2017), while others also reported comparable ERP responses (Batty and Taylor, 2006; Todd et al., 2008). Former studies mostly employed passive viewing tasks to discern differences in children’s facial expression processing, whereas our task asked children to actively match facial expressions, potentially influencing ERP responses. Thus, even though happy facial expressions seem to be most readily processed (De Sonneville et al., 2002), task demands might have led to a shift in saliency and attentional resources.

Our null results concerning the N170 are in line with some of the previous research, which did not report ERP modulations by happy or angry vs. neutral expressions in preschoolers (Todd et al., 2008; Dennis et al., 2009). The N170 component has been discussed to be more involved in facial structural encoding than in emotion detection, which is indicative of two parallel but independent stages of face processing (Bruce and Young, 1986). The N170 is also undergoing significant maturational changes (e.g., from bifid to unified trajectory; Batty and Taylor, 2006). Thus, the averaging process across children who show great N170 variability may have also diminished potential effects.

For late ERP responses, we detected larger P3 amplitudes for angry expressions as compared to happy or neutral expressions (effect also detected at Face 1, see Supplementary Tables S7, S9), which might suggest more in-depth analysis of negative facial expressions in young children. Firstly, emotions with negative valence, such as fear or anger, represent salient evolutionary value because they provide cues to retreat or prepare to defend oneself (Gao and Maurer, 2010). Therefore, larger amplitudes for angry vs. happy expressions may be indicative of a prioritization in processing potentially threatening stimuli (D'Hondt et al., 2017; Xie et al., 2019). Secondly, young children are less familiar with angry expressions, which has been shown in behavioral studies suggesting a protracted development of reliably detecting angry facial expression until later childhood (Gao and Maurer, 2010). Considering typical social environments during preschool time, expressions of anger might be quite novel and less frequent. An example of how environment shapes emotional development is discussed by one study who reported that children formerly exposed to high levels of parental anger recognized this emotion earlier than children of the control group (Pollak et al., 2009). These findings might explain why P3 amplitudes were largest for angry expressions. Regarding the null findings for P3 amplitude differences between happy vs. neutral expressions, one has to note that, before the age of nine, children often rate neutral faces as happy or sad (Durand et al., 2007). Thus, during in-depth face processing similar amplitudes might have been elicited for happy and neutral expressions.

### Repetition Effects in Early and Late ERPs

In line with our hypothesis, we detected reduced P1 amplitudes for repeated happy as compared to novel happy trials, which may be suggestive of decreased processing efforts and re-activations of existing memory traces particularly for happy expressions in young children (Nordt et al., 2016). In contrast, we did not find reduced P1 amplitudes for repeated angry or neutral as compared to novel angry or neutral expressions. Thus, neural representations for these facial expressions might not be as developed yet. This is in line with previous research indicating that happy expressions are most readily processed (Durand et al., 2007), whereas protracted trajectories have been found for angry or neutral expressions (Gao and Maurer, 2010). This ERP result is also paralleled by our reaction times findings of the EMT and delayed match-to-sample task indicating faster reaction times for pairings with happy expressions. In concordance, other studies also reported that the presence of happy faces facilitates the matching of emotion pairs (De Sonneville et al., 2002). Besides faster reaction times for happy vs. angry expressions in the delayed match-to-sample task, we also detected larger P1 amplitudes for novel happy as compared to novel angry expressions.

Alternatively, since we had stimulus contrast differences indicating larger contrast values for happy faces, it is possible that the P1 response was influenced by low-level stimulus differences. Previous studies indicated that the P1 is influenced by low-level stimuli changes (e.g., Rossion and Caharel, 2011; Schindler et al., 2021). There is, however, another research branch indicating that changes in low-level stimulus’ characteristics do not always suffice to cause P1 modulations (Dering et al., 2009, 2011). Recently, it was concluded that pure low-level accounts do not seem to fully explain P1 modulations in response to emotional stimuli, favoring accounts that propose a mixture of bottom-up and top-down processes to be indexed by the P1 (Klimesch, 2011; Bruchmann et al., 2020). Within an integrative review examining emotional face processing in ERPs, it was also argued that top-down and emotional bottom-up relevance do not act in isolation but can be regarded as interconnected phenomena (Schindler and Bublatzky, 2020). Additionally, we have controlled for stimulus’ differences by including both a random intercept for every stimulus and individual contrast values as covariate in order to minimize the impact of perceptual features on our effects.

While we detected first evidence at the level of the P1 for reduced processing efforts for repeated facial expressions, we found increased P3 amplitudes for repeated facial expressions compared to novel expressions, suggestive of increased efforts for repeated vs. novel faces. Previous studies indicated that these enhancements to repeated stimuli may be observed when a memory trace is being created (Peykarjou et al., 2016). Consequently, one could hypothesize that representations were built during their repeated presentation. This may in turn suggest that facial expression categories are not yet stable in young children and thus new exemplars of the same emotion category were added to children’s memory. Alternatively, behavioral studies suggested that the matching of similar compared to dissimilar facial representation requires highly demanding, effortful controlled information processing (De Sonneville et al., 2002; Watling and Damaskinou, 2018). Therefore, it may be that young children needed to activate more neural resources for matching repeated expressions (Vlamings et al., 2010). Both of these explanations add to the existing literature of the P3 indicating that this component can be associated with facial expression differentiation (Luo et al., 2010). In addition, the differential pattern of repetition for early and late neural stages suggest differential top-down and bottom-up processing of facial expressions apparent in young children (Schindler and Bublatzky, 2020).

As another alternative explanation, task demands might have been responsible for increased neural activity to repeated compared to novel expressions. Given that we presented a novel facial identity in every trial rather than keeping the identity constant across the whole experiment, it is possible that young children’s performance was hampered by the need to process two different facial dimensions (identity and emotion). This explanation might likely also account for the null findings for the N170 which has been associated both with the processing of facial identity (Schweinberger and Neumann, 2016) and the encoding of emotions (Hinojosa et al., 2015). In addition, a recent systematic review indicated that N170 effects were most frequently observed in passive viewing designs, indicating that concurrent task-dependent resources might have competed with resources of emotional decoding at this processing stage (Schindler and Bublatzky, 2020). However, we aimed to overcome this task load by increasing the typical face presentation time to ensure individual face discrimination (Peykarjou et al., 2016). Another study also provides evidence that performance in facial expression processing is similar to identity processing in young children (Johnston et al., 2011). However, we cannot exclude the possibility that their representation of Face 1 may have not been comprehensive enough to reduce processing efforts upon Face 2.

As suggested by previous studies, repetition effects might be enhanced by presenting several repetitions of the same face to build a stable stimulus’ representation (Campanella et al., 2002; Müller et al., 2013; He and Johnson, 2018). In contrast, participants in our study only saw a repeated face once which might have hampered repetition modulations. Another difference to former research is that we used longer than usual stimulus’ presentation times (Campanella et al., 2002; van Strien et al., 2011) to allow for complete encoding of the facial expressions (Peykarjou et al., 2016) given that young children’s face memory may not be as refined yet (Brenna et al., 2015). In addition, we employed a short ISI between Face 1 and Face 2 to decrease cognitive load (Mueller et al., 2020). Ultimately, we cannot exclude the possibility that cognitive load may have still been too high for our sample of young children. One also has to take into account that there might be higher heterogeneity in processing strategies as compared to adult samples (Cohen Kadosh et al., 2013). Clearly, further work is required to test these alternative possibilities, as well as to tease apart those task features that are critical for effects of emotion repetition.

### Associations Between Repetition Effects and Socio-Emotional Competence Measures

None of the correlations between P1 and P3 difference scores and emotion recognition or empathy scores were significant. Some of the previous research work examining preschoolers’ facial processing also indicated null findings when linking ERP responses with socio-emotional competencies (e.g., Vlamings et al., 2010; D'Hondt et al., 2017). Studies, which detected significant associations, mostly reported brain–behavior correlations in children from high-risk environments (Curtis and Cicchetti, 2011) or in clinical samples (e.g., autism spectrum conditions, Dawson et al., 2004). Since our sample consisted of neurotypical children from families with a middle to high socioeconomic status, variability of the data might have been too small to find effects. In addition, the study was powered for within-subject ERP effects, not for interindividual differences which—given the current sample—are bound to be rather small and thus not detectable by our correlational analysis. From a developmental perspective, young children only begin to understand other peoples’ emotions as well as differences between own and other’s emotions (Denham, 2018). Further, empathic skills within this age range are still maturing which might have also hampered effects. In line with previous research, it may also be possible that other socio-emotional competencies, such as emotion regulation, may be more susceptible to neural associations (Dennis et al., 2009). Thus, our null findings of associating the P1 and P3 with socio-emotional competence measures do not necessarily imply that presented ERP effects are not related to facial expression processing. Even if there would be truly no effect, the ERP components might still reflect the processing of emotional expressions; but indicating processes that are not susceptible to (subclinical) interindividual differences.

### Future Directions

With our study, we confirmed that the ability to recognize emotions from facial expressions differs depending on type of emotion in young children (Durand et al., 2007). To further understand the stability of perceptual boundaries between facial expression categories, future studies could use varying facial expression intensity to capture the threshold at which neural modulations by emotion as well as repetition are observable (Gao and Maurer, 2010). In parallel, assessments of arousal for different intensities could be collected to further examine the degree to which young children are aroused by facial expression stimuli and whether arousal levels are distinct between emotions (e.g., between positive or negative valences). This in turn could also add to the ongoing discussion about the discriminatory power of emotion categorization in comparison to a more dimensional approach (e.g., see Posner et al., 2005). Additionally, the ecological validity of the paradigm could be increased with more naturalistic and dynamic instead of static emotional facial expressions (Quadrelli et al., 2019), which would also allow studying the variability and context-dependency of emotions in childhood (LoBue and Ogren, 2021). Future research might also vary the number of repetitions or the degree of stimulus’ familiarity to disentangle potential effects of face identity and emotion. Some studies reported that repetition effects are greater for familiar than unfamiliar faces, so it might be useful to examine ERP effects with faces that preschoolers already know prior to the experiment (Henson, 2016). Regarding the relation of socio-emotional competences and ERPs, a broader range of both behavioral measures and variety in facial expressions could be examined to more specifically bridge the gap between neural correlates and concrete behavior. Further, it would be interesting to investigate modulations of ERP responses to facial expression regarding individual differences in children’s temperamental or personality traits.

### Limitations

To inform about limitations of general cognitive abilities or face memory in the developing brain, the paradigm could be tested within a longitudinal framework to look at improvement over time (Watling and Damaskinou, 2018). When comparing different age groups, it would be of special interest to examine whether there are changes in processing throughout the course of the experiment which could give insights about if and how a mental representation of a facial expression category is built (Nordt et al., 2016). Furthermore, one has to note that the number of trials for the emotion x repetition interaction was quite low. Our sample consisted of young children for whom it is challenging to go through very many trial repetitions. The small number of trials might have contributed decreases in signal-to-noise ratio which may in the future be addressed by examining fewer facial expressions with more trials. In addition, we observed slight amplitude modulations for the different experimental conditions starting already at baseline level. These differences may be also linked to decreases in signal-to-noise ratio, but could reflect potentially meaningful differences in emotion processing that should be also addressed in future research. Lastly, our sample consisted predominantly of upper/middle-class families. There is a need of children from low socioeconomic backgrounds to replicate findings in higher risk and more diverse samples (Bhavnani et al., 2021).

## Conclusion

The current study confirms that basic mechanisms of facial expression processing are already in place in young children. Within the age range targeted here, early perceptual processes seem to be predominantly activated when encoding differences between facial expressions. Paralleling previous behavioral findings, repetition effects were particularly apparent for happy facial expressions in early processing stages. In contrast, in-depth neural face processing was dominated by angry facial expressions. Further studies are warranted to determine the stability of facial expression processing throughout different developmental periods.

## Data Availability Statement

The datasets presented in this study can be found in online repositories. The names of the repository/repositories and accession number(s) can be found at: https://osf.io/nas48/.

## Ethics Statement

The studies involving human participants were reviewed and approved by Ethics committee of the Department of Psychology at Humboldt-Universität zu Berlin. Written informed consent to participate in this study was provided by the participants’ legal guardian/next of kin.

## Author Contributions

SN, MB, and ID: study design, interpretation of the data, and drafting and revising of the manuscript. SN: data collection and statistical analysis. All authors contributed to the article and approved the submitted version.

## Funding

This study was supported by funding from the Berlin School of Mind and Brain, Humboldt-Universität zu Berlin and the Stiftung der Deutschen Wirtschaft (sdw). We also acknowledge support by the German Research Foundation (DFG) and the Open Access Publication Fund of Humboldt-Universität zu Berlin.

## Conflict of Interest

The authors declare that the research was conducted in the absence of any commercial or financial relationships that could be construed as a potential conflict of interest.

## Publisher’s Note

All claims expressed in this article are solely those of the authors and do not necessarily represent those of their affiliated organizations, or those of the publisher, the editors and the reviewers. Any product that may be evaluated in this article, or claim that may be made by its manufacturer, is not guaranteed or endorsed by the publisher.
