# Supplementary material for: Preschoolers’ Sensitivity to Negative and Positive Emotional Facial Expressions: An ERP Study
Source: Front Psychol. 2022 May 31;13:828066. doi: 10.3389/fpsyg.2022.828066 (PMC9197498; doi:10.3389/fpsyg.2022.828066)

Preschoolers‘ sensitivity to negative and positive emotional facial expressions: An ERP study

Sandra Naumann[1, 2, *] | Mareike Bayer[1, 2] | Isabel Dziobek[1, 2]

[1] Berlin School of Mind and Brain, Humboldt-Universität zu Berlin, Berlin, Germany
[2] Department of Psychology, Institute of Life Sciences, Humboldt-Universität zu Berlin, Berlin, Germany
* Correspondence: sandra.naumann@hu-berlin.de

Supplementary material

Delayed match-to-sample-task *2*
TABLE S1 2
TABLE S2 2
ERP analyses 3
TABLE S3 3
TABLE S4 4
TABLE S5 4
TABLE S6 4
TABLE S7 5
TABLE S8 6
TABLE S9 6
Emotion recognition and empathy measures 7
TABLE S10 7
TABLE S11 7
TABLE S12 8
TABLE S13 8
Additional figures 9
FIGURE S1 9

Data availability: Data and code necessary to reproduce all analyses reported here, as well as additional supplementary files are available at https://osf.io/nas48/.

Delayed match-to-sample-task

TABLE S1 Results of the general linear mixed model (GLMM) used to test the hypotheses for accuracy rates and the linear mixed model used to test the hypotheses for reaction times (RT) of the delayed match-to-sample-task

	Accuracy				Reaction time			
Predictors	*Odds Ratios*	*SE*	*t*	*p*	*Estimates*	*SE*	*t*	*p*
Emotion (E) vs. Neutral (N)	1.00	0.11	0.03	0.974	0.05	0.05	1.01	0.311
Happy (H) vs. Angry (A)	0.85	0.08	-1.79	0.074	0.09	0.04	2.14	**0.032**
Repetition	0.99	0.08	-0.07	0.946	-0.02	0.03	-0.55	0.586
E vs. N x Repetition	1.25	0.27	1.05	0.294	-0.20	0.09	-2.18	**0.030**
H vs. A x Repetition	1.30	0.24	1.38	0.169	-0.11	0.08	-1.31	0.189
Working Memory	0.84	0.13	-1.14	0.255	0.06	0.06	1.00	0.319
**Random Effects**								
σ^2	3.29				0.71			
τ_{00}	0.03 $_{Stim\_Type}$				0.01 $_{Stim\_Type}$			
	0.60 $_{ID}$				0.10 $_{ID}$			
ICC	0.16				0.14			
N	28 $_{ID}$				28 $_{ID}$			
	72 $_{Stim\_Type}$				72 $_{Stim\_Type}$			
Marginal R^2 / Conditional R^2	0.010 / 0.169				0.009 / 0.144			

Note: p-values for the fixed effects calculated using Wald-statistics approximation, uncorrected. Model equations: Response ~ Emotion * Repetition + Working Memory + (1 | ID) + (1 | Stim_Type); RTs_log ~ Emotion * Repetition + Working Memory + (1 | ID) + (1 | Stim_Type). *SE*: standard error; *t*: test statistic coefficient; *p*: p-value; σ^2: within-group variance; $\tau 00$: between-group variance; *ICC*: interclass correlation (ratio of between-cluster variance to total variance); *N*: number of random effects.

TABLE S2 Post-hoc comparisons for Reaction times Emotional vs. Neutral x Repetition

Comparison						
condition		*condition*	*Est.*	*SE*	*z-value*	p_{FDR}
neutral $_{novel}$	-	angry $_{novel}$	0.04	.06	-0.72	.90
neutral $_{novel}$	-	happy $_{novel}$	0.18	.06	3.21	**.01**
neutral $_{repeated}$	-	angry $_{repeated}$	-0.06	.06	-1.00	.75
neutral $_{repeated}$	-	happy $_{repeated}$	-0.02	.06	-0.43	.98

Note. Est: estimates. *SE*: standard error. p_{FDR}: FDR-corrected p-values.

ERP analyses

TABLE S3 Results of the linear mixed models used to test the hypotheses for the facial expression and repetition effects at the P1, N170 and P3 component for Face 2

	P1 Amplitude				N170 Amplitude				P3 Amplitude			
Predictors	*b*	*SE*	*t*	*p*	*b*	*SE*	*t*	*p*	*b*	*SE*	*t*	*p*
Emotion (E) vs. Neutral (N)	1.29	0.40	-3.20	**0.001**	-0.08	0.38	-0.22	0.826	-1.05	0.45	-2.22	**0.020**
Happy (H) vs. Angry (A)	-0.27	0.35	-0.77	0442	0.11	0.33	0.32	0.749	1.10	0.39	2.84	**0.004**
Repetition	-0.11	0.33	-0.34	0.732	0.07	0.28	0.26	0.798	0.88	0.36	2.46	**0.014**
E vs. N x Repetition	1.20	0.81	1.49	0.137	0.87	0.77	1.14	0.256	0.73	0.90	0.81	0.420
H vs. A x Repetition	2.75	0.70	3.92	**<0.001**	1.16	0.66	1.76	0.079	-0.78	0.78	-0.99	0.321
Working Memory	1.05	0.97	1.08	0.278	0.78	0.63	1.24	0.217	0.38	1.23	0.31	0.760
Stimulus' Contrast	0.36	0.21	1.67	0.095	0.04	0.14	0.30	0.761	-0.50	0.23	-2.19	**0.029**
**Random Effects**												
σ^2	269.64				222.67				337.94			
τ_{00}	5.99 $_{Stim\_Type}$				0.19 $_{Stim\_Type}$				5.49 $_{Stim\_Type}$			
	26.54 $_{ID}$				10.79 $_{ID}$				43.24 $_{ID}$			
	8.99 $_{Elect\_site}$				1.57 $_{Elect\_site}$				3.46 $_{Elect\_site}$			
ICC	0.13				0.05				0.13			
N	28 $_{ID}$				28 $_{ID}$				28 $_{ID}$			
	72 $_{Stim\_Type}$				72 $_{Stim\_Type}$				72 $_{Stim\_Type}$			
	7 $_{Elect\_site}$				6 $_{Elect\_site}$				7 $_{Elect\_site}$			
Marginal R^2 / Conditional R^2	0.006 / 0.138				0.003 / 0.056				0.002 / 0.136			

Note: p-values for the fixed effects calculated using Wald-statistics approximation, uncorrected. Model equations: mean_ amplitude P1/ mean amplitude N170/ mean amplitude P3 ~ Emotion * Repetition + Working Memory + Stimulus' Contrast + (1 | ID) + (1 | Stim_Type) + (1 | Electrode). *SE*: standard error; *t*: test statistic coefficient; *p*: p-value; σ^2: within-group variance; $\tau 00$: between-group variance; *ICC*: interclass correlation (ratio of between-cluster variance to total variance); *N*: number of random effects.

TABLE S4 Post-hoc comparisons of P1 amplitudes for E vs. N contrast (Face 2)

Comparison						
condition		*condition*	*Est.*	*SE*	*z-value*	p_{FDR}
neutral	-	happy	-0.83	0.35	-2.38	**.033**
neutral	-	angry	-1.10	0.35	-3.18	**.003**

Note. Est: estimates. *SE*: standard error. p_{FDR} : FDR-corrected p-value.

TABLE S5 Post-hoc comparisons for P1 amplitude E vs. N x Repetition contrast (Face 2)

Comparison						
condition		*condition*	*Est.*	*SE*	*z-value*	p_{FDR}
happy $_{novel}$	-	angry $_{novel}$	1.64	0.49	3.33	**.005**
happy $_{novel}$	-	angry $_{repeated}$	0.68	0.50	1.35	.533
angry $_{repeated}$	-	angry $_{novel}$	0.96	0.52	1.86	.246
happy $_{repeated}$	-	angry $_{novel}$	-0.15	0.53	-0.27	.993
happy $_{repeated}$	-	happy $_{novel}$	-1.79	0.52	-3.44	**.003**
happy $_{repeated}$	-	angry $_{repeated}$	-1.11	0.50	-2.23	.115

Note. Est: estimates. *SE*: standard error. p_{FDR}: FDR-corrected p-value.

TABLE S6 Post-hoc comparisons of P3 amplitudes for E vs. N contrast (Face 2)

Comparison						
condition		*condition*	*Est.*	*SE*	*z-value*	p_{FDR}
neutral	-	happy	-0.24	0.39	-0.61	.767
neutral	-	angry	-1.34	0.39	-3.42	**.001**

Note. Est: estimates. *SE*: standard error. p_{FDR} : FDR-corrected p-value.

TABLE S7 Results of the linear mixed models for the P1, N170 and P3 component at Face 1

	**P1 Amplitude**				**N170 Amplitude**				**P3 Amplitude**			
Predictors	*b*	*SE*	*t*	*p*	*b*	*SE*	*t*	*p*	*b*	*SE*	*t*	*p*
Emotion vs. Neutral	-1.55	0.42	-3.72	**<0.001**	-0.79	0.40	-1.96	0.050	-2.18	0.49	-4.49	**<0.001**
Happy vs. Angry	0.34	0.37	0.93	0.352	1.05	0.36	2.94	**0.003**	0.96	0.43	2.23	**0.026**
Stimulus' Contrast	-0.28	0.21	-1.32	0.187	0.11	0.16	0.69	0.490	-0.20	0.26	-0.76	0.445
Working Memory	0.51	0.98	0.52	0.601	0.20	0.61	0.32	0.747	1.18	1.11	1.06	0.289
**Random Effects**												
σ^2	281.10				238.02				379.72			
τ_{00}	3.50 $_{Stim\_Type}$				0.63 $_{Stim\_Type}$				6.47 $_{Stim\_Type}$			
	26.96 $_{ID}$				10.20 $_{ID}$				34.77 $_{ID}$			
	6.54 $_{Elect\_site}$				3.52 $_{Elect\_site}$				4.53 $_{Elect\_site}$			
ICC	0.12				0.06				0.11			
N	28 $_{ID}$				28 $_{ID}$				28 $_{ID}$			
	72 $_{Stim\_Type}$				72 $_{Stim\_Type}$				72 $_{Stim\_Type}$			
	7 $_{Elect\_site}$				6 $_{Elect\_site}$				7 $_{Elect\_site}$			
Marginal R^2 / Conditional R^2	0.002 / 0.118				0.001 / 0.058				0.005 / 0.112			

Note: p-values for the fixed effects calculated using Wald-statistics approximation, uncorrected. Model equations: mean_ amplitude P1/ mean amplitude N170/ mean amplitude P3 ~ Emotion + Working Memory + Stimulus' Contrast + (1 | ID) + (1 | Stim_Type) + (1 | Electrode). *SE*: standard error; *t*: test statistic coefficient; *p*: p-value; σ^2: within-group variance; $\tau 00$: between-group variance; *ICC*: interclass correlation (ratio of between-cluster variance to total variance); *N*: number of random effects.

TABLE S8 Post-hoc comparisons of P1 amplitudes for E vs. N contrast (Face 1)

Comparison						
condition		*condition*	*Est.*	*SE*	*z-value*	p_{FDR}
neutral	-	happy	-0.91	0.36	-2.52	**.023**
neutral	-	angry	-1.34	0.36	-3.72	**< .001**

Note. Est: estimates. *SE*: standard error. p_{FDR} : FDR-corrected p-value.

TABLE S9 Post-hoc comparisons of P3 amplitudes for E vs. N contrast (Face 1)

Comparison						
condition		*condition*	*Est.*	*SE*	*z-value*	p_{FDR}
neutral	-	happy	-1.12	0.42	-2.66	**.015**
neutral	-	angry	-2.12	0.42	-5.04	**< .001**

Note. Est: estimates. *SE*: standard error. p_{FDR} : FDR-corrected p-value.

Emotion recognition and empathy measures

Emotion matching task (EMT)

TABLE S10 Results of the general linear mixed model and linear mixed model used to test the hypotheses for the accuracy rates and reaction times of the emotion matching task (EMT)

	Accuracy				Reaction time			
Predictors	*Odds Ratios*	*SE*	*t*	*p*	*Estimates*	*SE*	*t*	*p*
Emotional vs. Neutral	1.10	0.22	0.50	0.619	0.11	0.04	2.58	**0.010**
Happy vs. Angry	1.39	0.24	1.92	0.055	0.05	0.04	1.31	0.190
Working Memory	1.25	0.26	1.10	0.272	0.05	0.06	0.78	0.436
**Random Effects**								
σ^2	3.29				0.24			
τ_{00}	1.08 $_{Stim\_Type}$				0.00 $_{Stim\_Type}$			
	1.07 $_{ID}$				0.10 $_{ID}$			
ICC	0.40				0.30			
N	28 $_{ID}$				28 $_{ID}$			
	59 $_{Stim\_Type}$				59 $_{Stim\_Type}$			
Marginal R^2 / Conditional R^2	0.013 / 0.403				0.011 / 0.312			

Note: p-values for the fixed effects calculated using Wald-statistics approximation, uncorrected. Model equations: Response ~ Emo_Neu + Hap_Ang + Working Memory + (1 | ID) + (1 | Stim_Type); RT_log ~ Emo_Neu + Hap_Ang + Working Memory + (1 | ID) + (1 |Stim_Type). EMT: Emotion matching task. *SE*: standard error; *t*: test statistic coefficient; *p*: p-value; σ^2: within-group variance; $\tau 00$: between-group variance; *ICC*: interclass correlation (ratio of between-cluster variance to total variance); *N*: number of random effects.

TABLE S11 Post-hoc comparisons of reaction times for emotional vs. neutral contrast

Comparison						
condition		*condition*	*Est.*	*SE*	*z-value*	p_{FDR}
angry	-	neutral	-.06	.04	-1.6	.18
happy	-	neutral	-.10	.04	-2.9	**.01**

Note. Est: estimates. *SE*: standard error. p_{FDR} : FDR-corrected p-value.

ERP associations with socio-emotional competence measures

TABLE S12 Correlations between the standardized composite EMK 3-6 empathy (EM) score and P1 and P3 amplitude difference scores

	r	*t*	*p* uncorrected
EMK EM composite score	-	-	-
P1 Diff. happy $_{novel}$ – happy $_{repeated}$	-.15 [-.48, .21]	-0.84	.41
P1 Diff. happy $_{novel}$ – angry $_{novel}$	-.05 [-.40, .31]	-0.26	.79
P3 Diff. novel – repeated	-.12 [-.46, .24]	-0.66	.52

Note. Correlation coefficients were computed with Pearson‘s correlations. EMK 3-6 EM: Composite score for empathy of the *Inventory to survey of emotional competences for three to six-year-olds.* Values in square brackets indicate the 95% confidence interval for each correlation.

TABLE S13 Correlations between the standardized composite EMK 3-6 emotion recognition (ER) score and P1 and P3 amplitude difference scores

	r	*t*	*p* uncorrected
EMK ER composite score	-	-	-
P1 Diff. happy $_{novel}$ – happy $_{repeated}$	.06 [-.41, .30]	-0.34	.74
P1 Diff. happy $_{novel}$ – angry $_{novel}$	.01 [-.36, .35]	-0.04	.97
P3 Diff. novel – repeated	-.07 [-.42, .29]	-0.39	.70

Note. Correlation coefficients were computed with Pearson‘s correlations. EMK 3-6 EK: Composite score for emotion recognition of the *Inventory to survey of emotional competences for three to six-year-olds.* Values in square brackets indicate the 95% confidence interval for each correlation.

Additional figures

FIGURE S1. Topographies of the averaged A) P1 (90-130 ms), B) N170 (180-220 ms), C) P3 (300-500 ms) activity displaying difference topographies (in µV) for the emotion x repetition interaction.

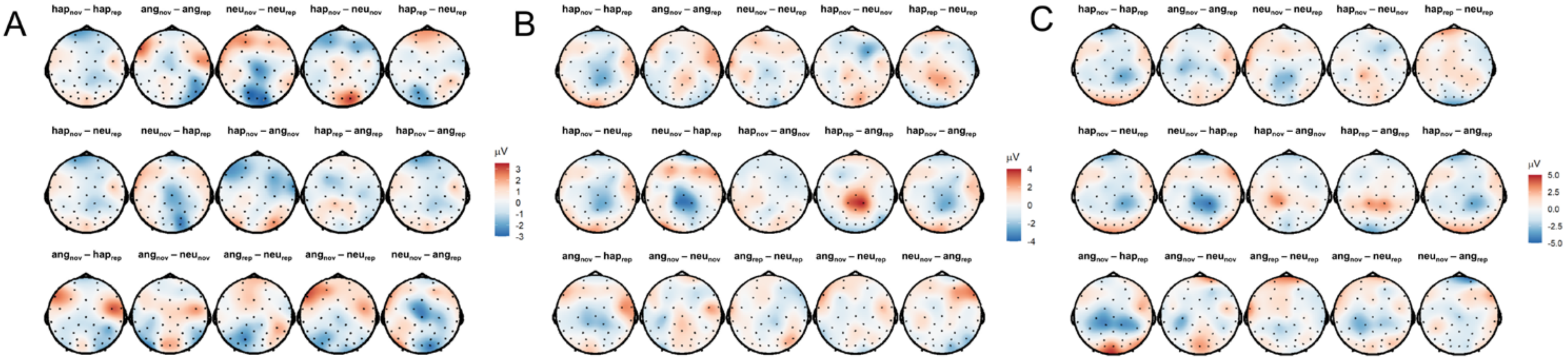

Supplement: Supplementary file 1 [file Data_Sheet_1.PDF]
